# Polymorph engineering of Cu*M*O_2_ (*M* = Al, Ga, Sc, Y) semiconductors for solar energy applications: from delafossite to wurtzite

**DOI:** 10.1107/S2052520615018387

**Published:** 2015-11-07

**Authors:** David O. Scanlon, Aron Walsh

**Affiliations:** a Department of Chemistry, University College London, England; b Diamond Light Source Ltd, Harwell Science and Innovation Campus, England; c Department of Chemistry, University of Bath, England; d Department of Materials Science and Engineering, Yousei University, Republic of Korea

**Keywords:** polymorphs, semiconductors, solar energy, structure–property relationships, first-principles materials modelling

## Abstract

The cuprous oxide based ternary delafossite semiconductors have been well studied in the context of *p*-type transparent conducting oxides. CuAlO_2_, CuGaO_2_ and CuInO_2_ represent a homologous series where the electronic properties can be tuned over a large range. The optical transparency of these materials has been associated with dipole forbidden transitions, which are related to the linear O—Cu—O coordination motif. The recent demonstration that these materials can be synthesized in tetrahedral structures (wurtzite analogues of the chalcopyrite lattice) opens up a new vista of applications. We investigate the underlying structure–property relationships (for Group 3 and 13 metals), from the perspective of first-principles materials modelling, towards developing earth-abundant photoactive metal oxides. All materials studied possess indirect fundamental band gaps ranging from 1 to 2 eV, which are smaller than their delafossite counterparts, although in all cases the difference between direct and indirect band gaps is less than 0.03 eV.

## Abstract 2

Structure–property relationships in Cu-based ternary oxides are explored using first-principles materials modelling.

## 1. Introduction   

Cu^I^ *M* ^III^O_2_ materials have been studied since 1873, when Friedel first discovered CuFeO_2_, and named the structure *delafossite* after the French crystallographer Gabriel Delafosse (Friedel, 1873 ▸). Since then, many delafossite structured compounds have been reported, including CuAlO_2_, CuGaO_2_, CuInO2_2_, CuScO_2_, CuYO_2_, CuCrO_2_, CuCoO_2_, CuLaO_2_ and CuNdO_2_, together with a number of cation mutated (cation cross substituted) quaternary oxides sharing the delafossite structure (Marquardt *et al.*, 2006 ▸). Interest in Cu-based delafossite structured oxides peaked in the last two decades, with the discovery of concomitant *p*-type conductivity and optical transparency in Cu*M*O_2_ (*M* = Al, Sc, Ga, In, Y, Ga; Kawazoe *et al.*, 1997 ▸; Ueda *et al.*, 2001 ▸) and more recently for their possible applications in photocatalysis (Gurunathan *et al.*, 2008 ▸). Poor conductivities and inefficient indirect band gaps have limited their applications as *p*-type transparent conductors (Scanlon & Watson, 2011*b* ▸; Tate *et al.*, 2009 ▸; Shin *et al.*, 2009 ▸). Conversely, poor optical absorption has limited their application in photocatalysis, despite the reasonable activity of CuCrO_2_ for water splitting (Saadi *et al.*, 2006 ▸; Arnold *et al.*, 2009 ▸; Scanlon *et al.*, 2009 ▸).

**Figure 1:**
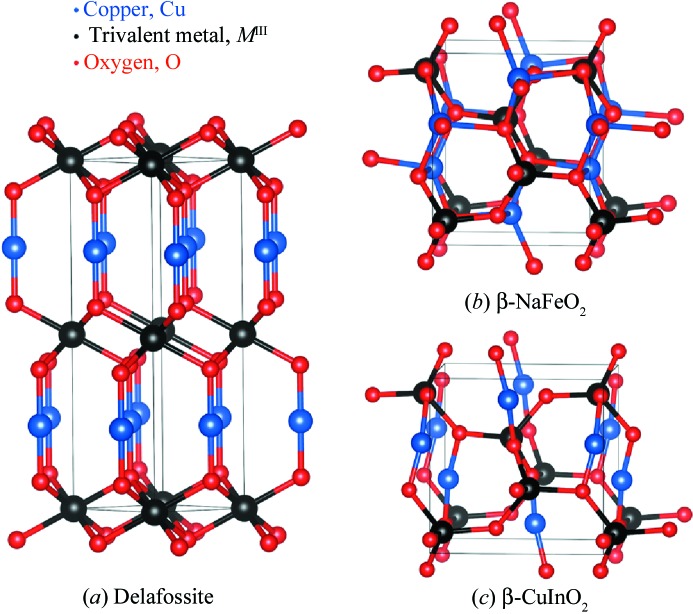
Representation of the crystal structure of (*a*) delafossite (hexagonal setting), (*b*) β-NaFeO_2_ and (*c*) the calculated β-CuInO_2_ structure. Note that β-NaFeO_2_ is isostructural to BeSiN_2_ and the parent of the hexagonal kesterite and stannite structures (Chen *et al.*, 2010 ▸).

In the delafossite structure each Cu atom is linearly coordinated with two O atoms, forming O—Cu—O dumbbells parallel to the *c* axis; see Fig. 1 ▸(*a*). O atoms in these O—Cu—O units are also each coordinated to three Al atoms, oriented such that Al-centred octahedra form AlO_2_ layers which lie parallel to the *ab* plane. Alternative layer stackings are possible, resulting in a hexagonal (space group *P*6_3_/*mmc*) or rhombohedral (space group 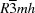) unit cell (Köhler & Jansen, 1986 ▸).

In 2014, however, CuGaO_2_ crystallizing in the ortho­rhombic β-NaFeO_2_ structure was reported (Fig. 1 ▸ *b*) and was shown to possess an optical band gap of ∼ 1.5 eV (Omata *et al.*, 2014 ▸). The synthesis was achieved by an ion exchange process starting from a β-NaFeO_2_ precursor. This direct gap material possesses a band gap that would indicate a maximum efficiency of ∼ 33% according to the Shockley–Queisser detailed balance limit (Shockley & Queisser, 1961 ▸). A small band gap oxide absorber has long been sought after by the photovoltaic community (Lee *et al.*, 2014 ▸).

In this paper we investigate computationally the geometry, stability and electronic structure of a family of β-NaFeO_2_ structured Cu*M*O_2_ (*M* = Al, Ga, In, Sc, Y, La) using a screened hybrid-density functional theory approach. We demonstrate:

(i) β-CuGaO_2_ is an indirect band gap semiconductor with a 1.0 eV fundamental band gap,
(ii) the optical band gaps of these β-CuMO_2_ compounds is greater than their fundamental band gaps due to a very weak onset of absorption and
(iii) the tetrahedral coordination of the Cu ions leads to a reduced mixing between the Cu 3*d* states and the O 2*p* states at upper valence band, producing a localized valence band maximum (VBM) of Cu 3*d* states.

The implications of this unusual electronic structure compared with delafossite oxides is discussed.

## 2. Computational methods   

All total energy and electronic structure calculations were performed within density functional theory (DFT) and periodic boundary conditions as implemented in the code *VASP* (Kresse & Furthmüller, 1996 ▸). Interactions between the core and valence electrons were described within the projector augmented wave method (Kresse & Joubert, 1999 ▸). The calculations were performed using the PBE (Perdew *et al.*, 1996 ▸) exchange–correlation functional augmented with 25% screened non-local Hartree–Fock electron exchange, producing the hybrid HSE06 functional (Krukau *et al.*, 2006 ▸). HSE06 has been successfully utilized to reproduce improved structural and band gap data compared with ‘standard’ local and semi-local DFT exchange–correlation functionals for many oxide semiconductors (Kehoe *et al.*, 2011 ▸; Scanlon *et al.*, 2011 ▸; Scanlon & Watson, 2011*a* ▸,*b* ▸; Allen *et al.*, 2010 ▸; Henderson *et al.*, 2011 ▸). Here the primary role of the Hartee–Fock exchange is the cancellation of the artificial self-interaction that arises from the mean-field treatment of the Coulomb interaction between electrons.

A planewave cutoff of 750 eV and a *k*-point sampling of 6 × 6 × 6 for the 12 atom unit cell of β-CuGaO_2_ were used, with the ionic forces converged to less than 0.01 eV Å^−1^. The optical transition matrix elements, calculated following Fermi’s golden rule, were used to construct the imaginary dielectric function and the corresponding optical absorption spectrum (Gajdoš *et al.*, 2006 ▸).

## 3. Results   

### 3.1. Crystal structure   

The calculated structural data for β-Cu*M* ^III^O_2_ is displayed in Table 1 ▸. The equilibrium structure for β-CuGaO_2_ is in excellent agreement with that of the recent experimental report (Omata *et al.*, 2014 ▸). For the rest of the family the data looks reasonable, except for β-CuYO_2_, β-CuInO_2_ and β-CuLaO_2_. All seven materials crystallize in the space group 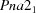, but due to the large cationic radius of Y, In and La the oxygen coordination sites in these systems deviate significantly from tetrahedral. In β-CuYO_2_ and β-CuLaO_2_ the O atoms remain four-coordinate, but close to a pyramidal coordination. In the case of β-CuInO_2_, upon relaxation the system is spontaneously distorted to form linear O—Cu—O dumbells, as shown in Fig. 1 ▸(*c*). Similar coordination is seen in other Cu^I^-containing oxides such as Cu_2_O, PbCu_2_O_2_ and SrCu_2_O_2_ (Godinho *et al.*, 2008 ▸, 2010 ▸; Modreanu *et al.*, 2007 ▸; Nolan, 2008 ▸; Scanlon & Watson, 2011*a* ▸).

We have also calculated the difference in enthalpy between the delafossite and β-Cu*M* ^III^O_2_, as shown in Table 1 ▸. In each case the delafossite is more stable than the β-Cu*M* ^III^O_2_ structure, although this is not necessarily a barrier to the formation of the β-Cu*M* ^III^O_2_ phase, as the synthesis method (ion exchange) is kinetically limited rather than thermodynamically controlled.

### 3.2. Electronic structure   

**Figure 2:**
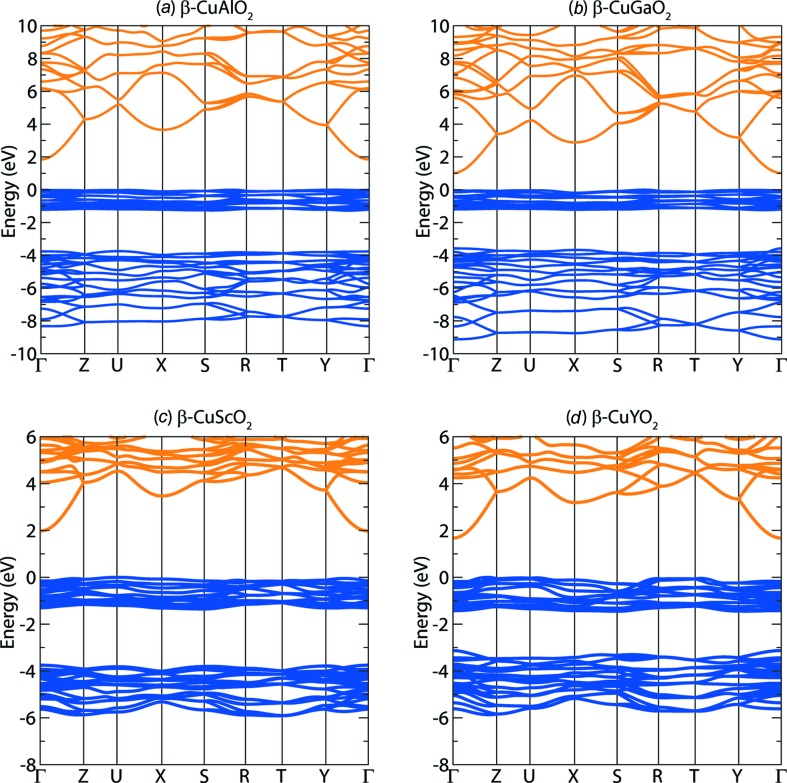
The hybrid DFT (HSE06) calculated electronic band structures for (*a*) β-CuAlO_2_, (*b*) β-CuGaO_2_, (*c*) β-CuScO_2_ and (*d*) β-CuYO_2_.

The calculated electronic band structures for β-CuAlO_2_, β-CuGaO_2_, β-CuScO_2_ and β-CuYO_2_ crystal structures are displayed in Fig. 2 ▸. For the Group 13 series, the band gap trend is Al > Ga < In, and for the Group 3 series the band gap trend is Sc > Y < La. In both cases In and La can be considered outliers. The reducing band gap down the groups is initially maintained, similar to the case of the Group 3 and 15 delafossites (Huda *et al.*, 2009*a* ▸). For all cases, the conduction band minimum (CBM) shows reasonable dispersion in reciprocal space, with the VBM being extremely flat (high hole effective mass). Localized flat bands appear for 1 eV below the VBM, and then a 2 eV gap appears to 4 eV of more localized electronic states.

**Figure 3:**
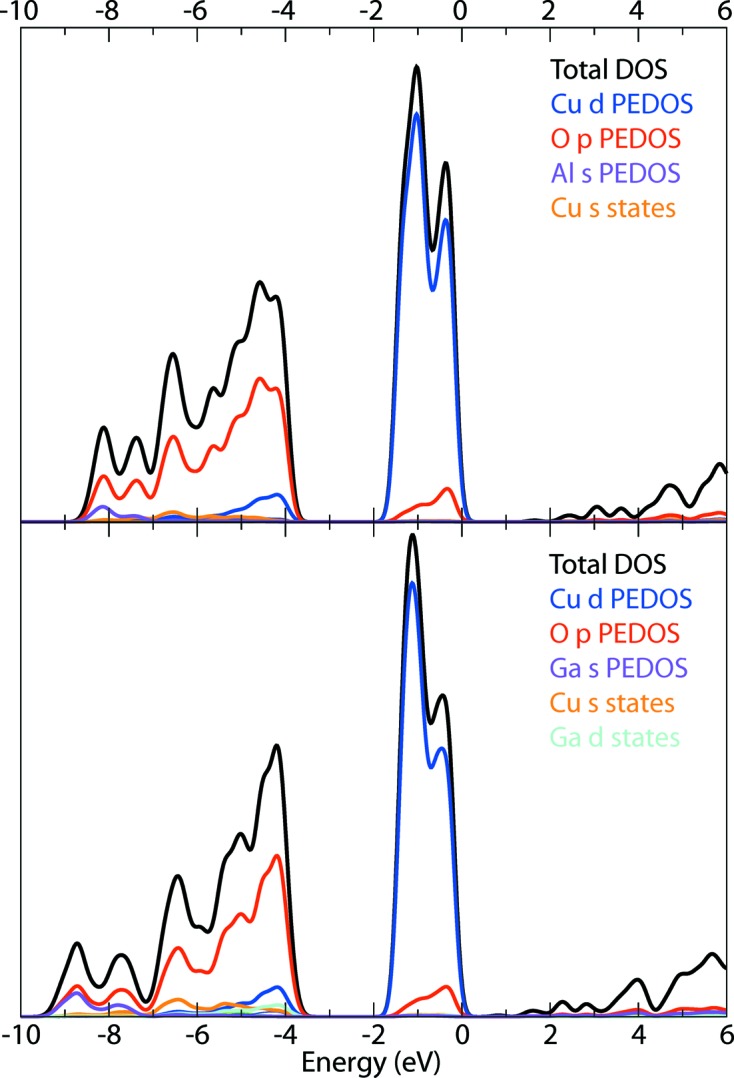
The hybrid DFT (HSE06) calculated electronic density of states for (upper panel) β-CuAlO_2_ and (lower panel) β-CuGaO_2_. The atomic components are obtained by projecting the periodic wavefunctions onto atom-centred spherical harmonics.

Analysis of the partial electronic densities of states (Fig. 3 ▸) reveals that the upper valence band is dominated by Cu 3*d* states, with little mixing between the O 2*p* and Cu 3*d* states. In fact, the O 2*p* states are separated from the Cu 3*d* states by ∼ 2 eV. This is not consistent with the chemical bonding of the delafossite structured Cu*M*O_2_ materials (Wei *et al.*, 1992 ▸). The conduction bands are dominated by *M* ^III^ *s* states for the Group 3 and 13 cations. This is unusual, as the *M* *d* states dominate the lower conduction band for the delafossite-structured CuScO_2_ and CuYO_2_.

### 3.3. Optical response   

**Figure 4:**
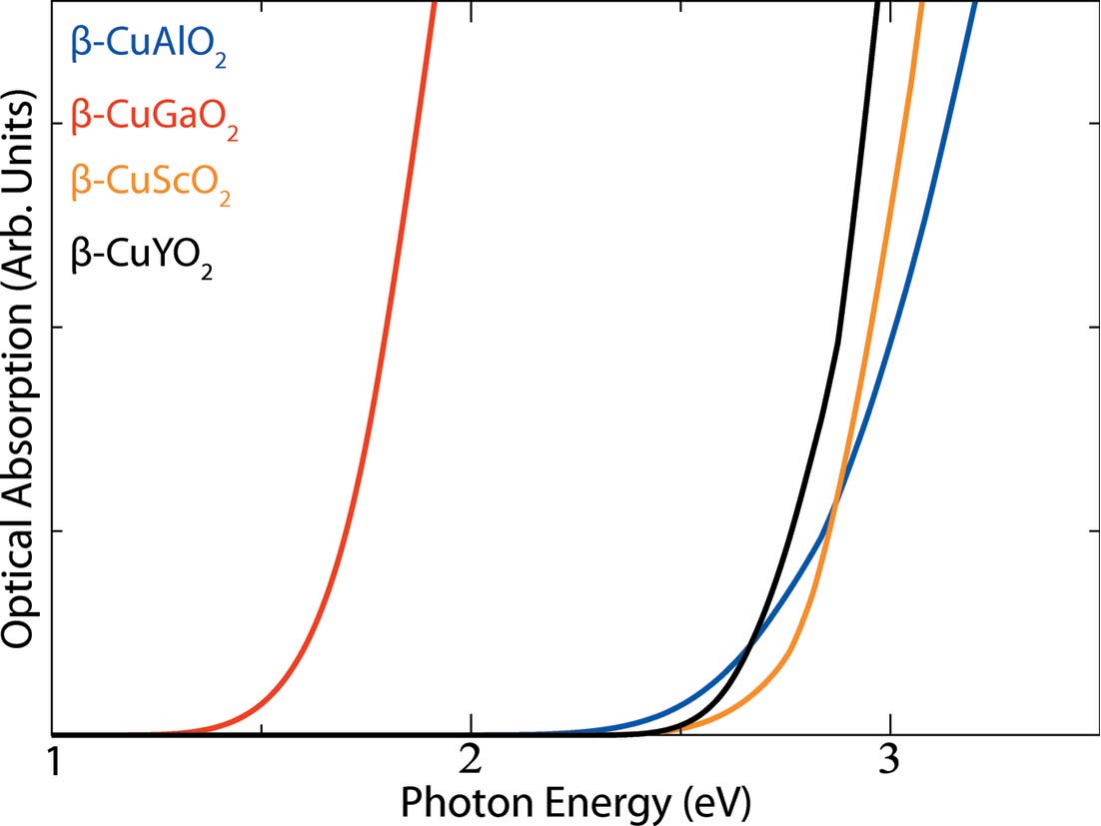
Predicted optical absorption onsets of β-CuAlO_2_, β-CuGaO_2_, β-CuScO_2_ and β-CuYO_2_ derived from the dielectric functions computed using density functional theory.

We have further calculated the optical absorption spectra, in the single-particle regime using Fermi’s Golden rule, with the results presented in Fig. 4 ▸. For all materials, the optical band gap is considerably larger than the fundamental electronic band gap. The simulated optical band gap for β-CuGaO_2_ is ∼ 1.5 eV, in excellent agreement with the experimental measurements (Omata *et al.*, 2014 ▸). To understand the differences between the *fundamental* indirect band gap and the direct allowed *optical* band gap, we have analysed the transition matrix elements for the allowed valence to conduction band transitions. Transitions from the VBM to CBM at the Γ point (*k* = 0,0,0) are dipole allowed; however, they are negligible until ∼ 0.5 eV higher in energy. This is due to the change in angular momentum of the bands (from *d* to metal *s* character orbitals). β-CuGaO_2_ has the smallest band gap with β-CuAlO_2_ possessing the largest optical band gap of ∼ 2.5 eV.

## 4. Discussion and conclusion   

The vastly different electronic structures exhibited by the delafossite and wurtzite materials can be explained by considering the role of the coordination of the Cu states in these systems.

Cu^I^ has the *d* ^10^ electronic configuration. The isolated ion is well known to have low lying *d* ^9^ *s* ^1^ excited states, which can mix into the ground state in a crystal environment if the site symmetry allows (Orgel, 1958 ▸). The common linear coordination preference of the cuprous ion has long been attributed to 3*dz* ^2^ − *s* hybridization, which compensates for a low coordination number. In the delafossite structure, there is effective energetic and spatial overlap of the O 2*p* and Cu 3*dz* ^2^ + *s* hybrid orbitals, resulting in large valence band dispersion and light hole masses.

In the tetrahedrally coordinated β phases, the same mixing is not achievable. The stronger anion field around the Cu atoms destabilizes the 3*d* band, which is split off in energy from the O 2*p* states. The result is a localized valence band with a large hole effective mass. Since the delafossites are known to be good *p*-type semiconductors, and the conduction band dispersion of wurtzite structured materials is likely to give rise to effective *n*-type conductivity, their combination could be used to form all-oxide *p*–*n* junctions. Such heterojunctions may be formed of one chemical composition in two structural forms.

These new insights into the electronic structure of β-CuGaO_2_ and related materials, however, are not entirely promising for the future use of this material for solar cell applications. The large difference in the electronic and optical band gaps will limit the open circuit voltage, and the localized states at the valence band maximum will likely limit carrier transport and collection. It is possible that the electronic structure could be tuned by alloying with β-CuAlO_2_ (the combination of different sizes on the *M* ^III^ site could make the weak transitions from the valence to conduction bands stronger, as was proposed previously for delafossite alloys; Huda *et al.*, 2009*b* ▸). Furthermore, the high dispersion in the conduction bands emphasizes the possibly of robust *n*-type conductivity, if a suitable *n*-type dopant was found.

In summary, polymorph engineering can produce unexpected effects in the electronic structure of multi-component materials. The kinetic control of crystallization products may reveal new phases with novel properties from well known materials systems.

## Figures and Tables

**Table 1:** DFT/HSE06 calculated lattice parameters and bond lengths in β-Cu*M* ^III^O_2_ (*M* = Al, Ga, In, Sc, Y, La), and energy difference between the delafossite and β phases A positive number indicates that the β phase is less stable than the delafossite phase.

System	*a* (Å)	*b* (Å)	*c* (Å)	Δ*H*_f_ (eV per atom)
β-CuAlO_2_	5.29	6.46	5.21	0.146
β-CuGaO_2_	5.46	6.63	5.29	0.119
β-CuGaO_2_ (Omata *et al.*, 2014 ▸)	5.46	6.61	5.27	–
β-CuInO_2_	6.55	6.61	6.46	0.228
β-CuScO_2_	5.92	6.58	5.42	0.291
β-CuYO_2_	6.53	6.75	5.26	0.359
β-CuLaO_2_	6.77	6.85	5.26	0.327
